# Pyroptosis for osteoarthritis treatment: insights into cellular and molecular interactions inflammatory

**DOI:** 10.3389/fimmu.2025.1556990

**Published:** 2025-04-01

**Authors:** Minghui Lin, Cunxin Zhang, Haiming Li, Kang Li, Shuao Gou, Xiao He, Chaoliang Lv, Kai Gao

**Affiliations:** ^1^ Second College of Clinical Medicine, Shandong University of Traditional Chinese Medicine, Jinan, China; ^2^ Department of Orthopedics, Jining No.1 People’s Hospital, Jining, China; ^3^ Jining No.1 People's Hospital, affiliated with Shandong First Medical University and Shandong Academy of Medical Sciences, Jinan, China; ^4^ Medical Integration and Practice Center, Shandong University, Jinan, China

**Keywords:** pyroptosis, osteoarthritis, chondrocytes, synoviocytes, NLRP3, inflammatory cytokines

## Abstract

Osteoarthritis (OA) is a widely prevalent chronic degenerative disease often associated with significant pain and disability. It is characterized by the deterioration of cartilage and the extracellular matrix (ECM), synovial inflammation, and subchondral bone remodeling. Recent studies have highlighted pyroptosis—a form of programmed cell death triggered by the inflammasome—as a key factor in sustaining chronic inflammation. Central to this process are the inflammatory cytokines interleukin-1β (IL-1β) and interleukin-18 (IL-18), which play crucial roles mediating intra-articular pyroptosis through the NOD-like receptor protein 3 (NLRP3) inflammasome. This paper investigates the role of the pyroptosis pathway in perpetuating chronic inflammatory diseases and its linkage with OA. Furthermore, it explores the mechanisms of pyroptosis, mediated by nuclear factor κB (NF-κB), the purinergic receptor P2X ligand-gated ion channel 7 (P2X7R), adenosine monophosphate (AMP)-activated protein kinase (AMPK), and hypoxia-inducible factor-1α (HIF-1α). Additionally, it examines the interactions among various cellular components in the context of OA. These insights indicate that targeting the regulation of pyroptosis presents a promising therapeutic approach for the prevention and treatment of OA, offering valuable theoretical perspectives for its effective management.

## 1 Introduction

Osteoarthritis (OA) is a widely prevalent degenerative disease characterized by the deterioration of intra-articular tissues and cells, frequently accompanied by inflammation. Epidemiological data indicate that approximately 240 million individuals globally suffer from OA (1). The incidence of OA escalates with age, and women are particularly more susceptible, experiencing higher rates and earlier onset compared to men, which imposes a significant economic burden on individuals and society (2, 3). OA manifests symptoms such as joint pain, swelling, stiffness, and restricted movement (4). Over time, the expansion of local inflammatory processes within the joint leads to structural changes in the articular cartilage, synovial tissues, and subchondral bone (5). Significantly affecting patients’ lives of through pain, OA is a primary cause of reduced mobility and diminished quality of life among the elderly, highlighting the need for more effective prevention and treatment strategies (6). Current treatments primarily focus on symptom relief rather than disease prevention. Conservative treatment options include pharmacotherapy and non-drug interventions such as education, self-management, exercise, and weight management (7). However, while pharmacotherapy may temporarily alleviate pain, it can also lead to adverse reactions such as gastrointestinal bleeding, edema, hypertension, and heart failure, potentially hastening the need for joint replacement surgery (8, 9). Moreover, there is a lack of medications that effectively prevent joint synovial inflammation, which can promote cell death in joint tissues and contribute to the progression of OA. Therefore, understanding the underlying mechanisms of OA and developing effective treatment strategies are urgently needed.

The pathogenesis of OA is multifaceted, characterized by irreversible cartilage destruction and uncontrolled chronic inflammation. Current research focuses intensely on deciphering the complex mechanisms behind inflammation-mediated cell death in OA joint tissue, aiming to develop targeted and effective therapeutic interventions that curb this pathogenic process (10). However, the specific mechanisms and therapeutic targets related to inflammation and histocyte dysfunction remain elusive, with a notable deficiency in targeted therapies to halt OA progression. Recent studies have highlighted that the overactivation of pyroptosis, a type of programmed cell death mediated by inflammasomes, plays a significant role in chronic aseptic inflammation associated with OA (11–13). Additionally, mounting evidence suggests that pyroptosis frequently triggers excessive inflammation and secondary damage to essential tissues, leading severe infections and exacerbating the progression of various diseases, including neuroinflammatory, cardiovascular, and inflammatory diseases affecting vital organs (14–16). Consequently, anti-inflammation interventions that effectively modulate the pyroptosis pathway have shown considerable promise as a strategic approach to treating treatment of inflammatory diseases, notably in the case of OA.

This review synthesizes the fundamental molecular mechanisms controlling pyroptosis, examining the intricate signaling cascades that initiate this process. Special attention is given to pyroptosis in the context of OA, detailing current research advancements related to the primary intra-articular tissues such as the synovium, cartilage, and subchondral bone. We also investigate the impact of these signaling pathways on OA progression, with the ultimate goal of improving therapeutic and management strategies for the disease.

## 2 Pyroptosis

### 2.1 Research process of pyroptosis

Pyroptosis was initially identified in macrophages of mice infected with Salmonella typhi, which was initially mistaken for apoptosis (17). Subsequent research by Hilbi et al. (18) demonstrated that mice lacking cysteine-aspartic acid protease-1 (Caspase-1) and infected with Shigella flexneri did not exhibit apoptosis in their macrophages. This discovery prompted a reevaluation of the distinctions between apoptosis and pyroptosis. Further investigations by Bergsbaken et al. (19) revealed that Salmonella could induce rapid macrophage death, attributed to Caspase-1-mediated cytotoxicity, thus defining this process as pyroptosis. Additional studies have established a significant link between the Caspase family and pyroptosis, with findings by Matikainen et al. (20) indicating that Caspase-4/5/11 are also involved in this cell death process. Notably, Caspase-11 indirectly influences interleukin-1β (IL-1β) through the Caspase-1 pathway (21), suggesting a regulatory interplay among Caspases in pyroptosis.

Pyroptosis is a distinct form of programmed cell death, differentiated from apoptosis and necrosis. It is characterized by inflammasome activation and the release of pro-inflammatory cytokines such as IL-1β and interleukin-18 (IL-18). In contrast, apoptosis is driven by apoptotic proteins such as Caspase-3, and necrosis typically results from external insults such as ischemia or toxins (22–24). Pyroptosis leads to cell swelling, lysis, and membrane rupture, which release cellular contents that provoke inflammation. Although apoptosis and pyroptosis share certain features, pyroptosis is uniquely inflammatory and regulated. Apoptosis, on the other hand, involves cell shrinkage, nuclear condensation, DNA fragmentation, and apoptotic body formation, all while maintaining membrane integrity (25–27). Pyroptosis plays a crucial role in combating infections and managing immune responses; apoptosis helps maintain tissue homeostasis by removing damaged or unnecessary cells; and necrosis is an uncontrolled response to cell injury (28). Since its discovery, this uniquely programmed Caspase activity-dependent pro-inflammatory process has attracted significant attention, particularly following the identification of pyroptosis in innate immune cells, highlighting its critical biological significance (29). Moreover, recent evidence suggests that pyroptosis also occurs in non-immune cells. As a key component of innate immunity, pyroptosis is essential in defending against infections and alarmins. It is widely implicated in the development and progression of tumors (30), infectious diseases (31), and degenerative inflammatory disorders (32).

Notably, pyroptosis appears to function as a double-edged sword within histocytes. During the inflammatory phase, moderate pyroptosis inhibits intracellular pathogen replication, removes damaged cells, and induces a controlled inflammatory response to counteract danger signals (33). However, excessive pyroptosis can lead to widespread cellular destruction, ultimately exacerbating the progression of multiple diseases (34). Consequently, increasing attention has been directed toward the role of pyroptosis, with growing expectations that its regulation may serve as a pivotal therapeutic strategy in managing inflammatory diseases.

### 2.2 Pathways involved in pyroptosis

**Figure 1:**
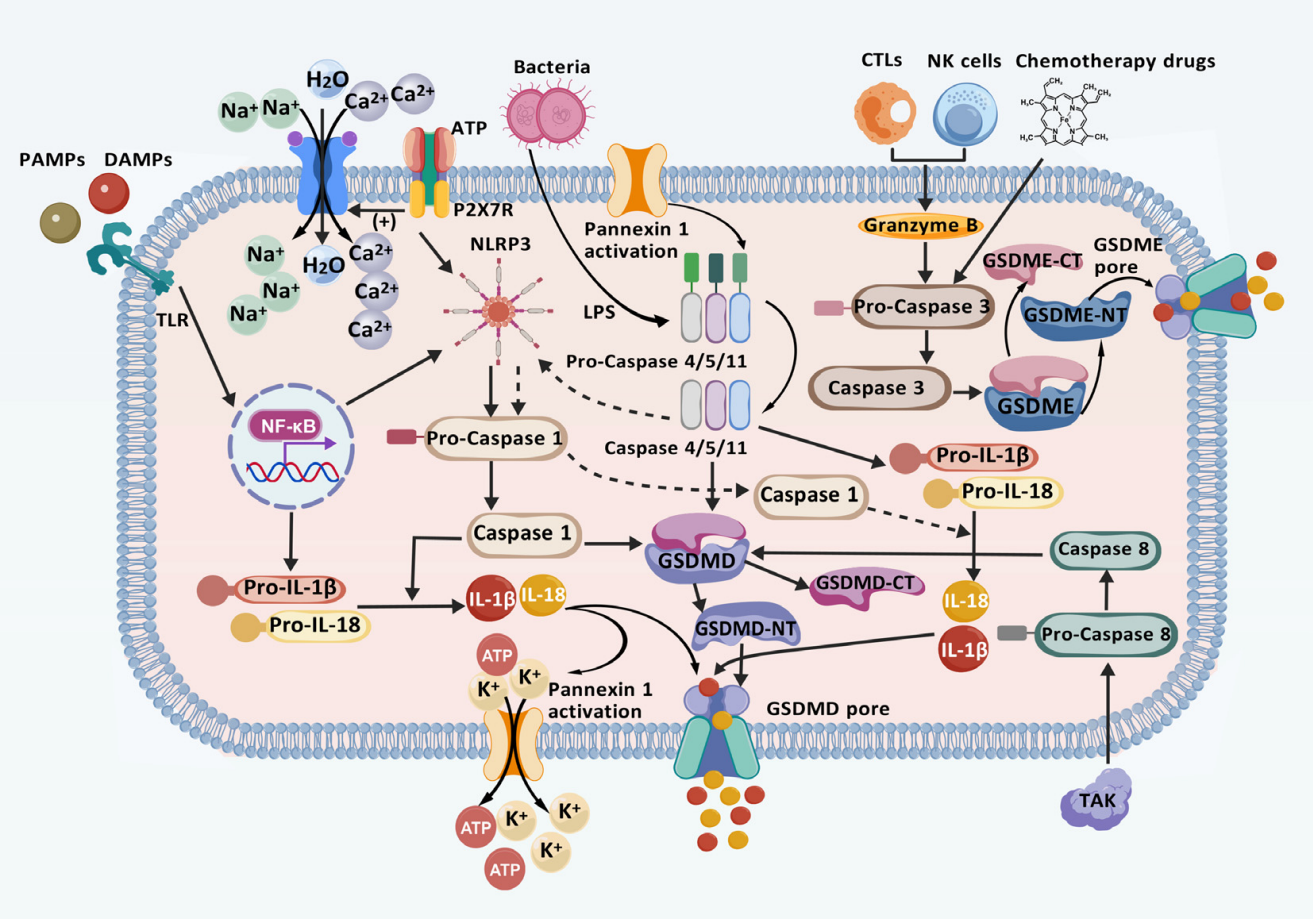
Mechanisms of classical, non-classical, and other pathways involved in pyroptosis. In the classical pathway, PRRs detect intracellular and extracellular signals, initiating the NF-κB/NLRP3 signaling cascade and activating the inflammasome. This activation leads to the cleavage of Caspase-1, which cleaved GSDMD. GSDMD then aggregates and inserts into the plasma membrane to form a membrane pore. Subsequently, Caspase-1 facilitates the release of inflammatory factors such as IL-1β and IL-18, promoting ion flow, water influx, cell swelling, and eventual cell rupture through the GSDMD pore, leading to pyroptosis. In the atypical pathway, LPS activates Pro Caspase-4/5/11, promoting the production of IL-1β and IL-18, production and inducing pyroptosis. Furthermore, Caspase-11 activation leads to intracellular K^+^ efflux and ATP release through the cleavage and modification of Pannexin-1. When ATP binds to P2X7R, it opens Ca^2+^ and Na^+^ influx channels, leading to rapid depolarization and pyroptosis. Additionally, in other pathways, TKA1 enhances Caspase-8 activation, initiating the GSDMD pore formation. Similarly, CTLs, NK cells, and the chemotherapeutic agent Caspase-3 activate the GSDME pore. These processes promote the production of inflammatory factors that ultimately contribute to pyroptosis. (Created with BioGDP.com).

Pyroptosis is a programmed inflammatory form of cell death initiated by the inflammasome and executed by the gasdermin family (22). The inflammasome is a cytoplasmic multiprotein complex composed of pattern recognition receptors (PRRs), apoptosis-associated speck-like protein containing a Caspase recruitment domain (CARD) (ASC), and Caspase-1. It plays a crucial role in detecting and responding to both exogenous pathogens and endogenous danger signals (35). ASCs serves as an adaptor protein, bridging sensor proteins with effector components (36). Gasdermins (GSDMs), including GSDM (A–E) and Pejvakin, constitute a family of membrane pore-forming proteins (37). Among these, gasdermin D (GSDMD) is the primary executor, possessing the ability to initiate pore formation, disrupt cell membranes, and release inflammatory cytokines, while Caspases are crucial components (38, 39). Based on the type of Caspase involved, pyroptosis is classified into classical, non-classical, and other alternative pathways (40) (**Figure 1**).

#### 2.2.1 The classical pathway of pyroptosis

In the classical activation pathway, the activation of NOD-like receptor thermal protein domain- associated protein-3 (NLRP3) is regulated by pathogen-associated molecular patterns (PAMPs) or damage-associated molecular patterns (DAMPs) (41). The activation process of NLRP3 occurs in two distinct phases: “activation” and “assembly”. Although the precise mechanisms underlying NLRP3-induced pyroptosis remains unclear, it is widely hypothesized that the formation of inflammasome vesicles plays a central role in the activation process, ultimately leading to Caspase-1 (42). NLRP3 activation is triggered by the exposure to various activators, including microparticles, toxins, adenosine triphosphate (ATP), pathogens, crystals, and protein aggregates, which induce the accumulation of mitochondrial reactive oxygen species (ROS) (43). The activation process of NLRP3 involves two steps: “activation” and “assembly”. The activation phase begins when PAMPs bind to Toll-like receptors (TLRs), activating the nuclear factor-κB (NF-κB) pathway (44). This process is further modulated by regulatory non-coding RNAs, including circular RNAs (circRNAs) and microRNAs (miRNAs). Specifically, miR-665 has been identified as a key regulator of circRNF121 and myeloid differentiation primary response protein 88 (MyD88) (44, 45). Through the circRNF121/MyD88/NF-κB signaling pathway, miR-665 promotes the transcription of downstream genes, including NLRP3, Caspase family proteins, interleukin-1β precursor (pro-IL-1β), and interleukin-18 precursor (pro-IL-18), leading to the production of these inflammatory mediators (45, 46). During the execution phase, the assembly of NLRP3 depends on mitochondrial DNA (mtDNA), as an increase in advanced oxidation protein products (AOPP) leads to a decline in mitochondrial membrane potential, thereby elevating mitochondrial reactive oxygen species (mtROS). This increase in mtROS oxidizes, releasing oxidized mitochondrial DNA (Ox-mtDNA), which binds to cytosolic NLRP3, ultimately triggering inflammasome assembly (47–49). Cytidine/uridine monophosphate kinase-2 (CMPK2) is an enzyme that promotes mtDNA synthesis, resulting in the formation of Ox-mtDNA fragments, and its activity is regulated in an interferon regulatory factor-1 (IRF1)-dependent manner (50, 51). Once the inflammasome is assembled, NLRP3 oligomerization occurs through interactions among its homologous Nacht domains, forming a high-molecular-weight complex that induces Caspase-1 autoactivation (35). Caspase-1 activation subsequently cleaves its precursor, leading to the release and activation of Caspase-1. This enzyme then converts the pro-IL-1β and pro-IL-18 precursors into their mature cytokine forms, IL-1β and IL-18, through proteolysis (52). Concurrently, GSDMD remains inhibited, with its N-terminal and C-terminal domains directly linked (53). During the process of pyroptosis, the Caspase-1-mediated cleavage at ASP275 initiate the proteolysis of GSDMD, producing a 31 kDa GSDMD-NT fragment and a 22 kDa GSDMD-CT fragment (54). The GSDMD-NT protein binds to acidic phospholipids on the cell membrane, forming a pore composed of 16 identical proteins. This pore formation disrupts the ion concentration balance across the cell membrane, causing water influx, cell swelling, and the leakage of cellular contents (39). Ultimately, cell lysis occurs, releasing inflammatory mediators such as IL-1β, IL-18, and high mobility group box 1 (HMGB1), which stimulate the inflammatory response and contribute to pyroptosis (55).

#### 2.2.2 Non-classical pyroptosis pathway

The non-canonical pathway pyroptosis primarily involves human Caspase-4/5 and their murine counterparts, Caspase-11 (39). Lipopolysaccharides (LPS), either intracellular or from Gram-negative bacteria, binds to the CARD domain of Caspase-4/5/11, thereby activating these Caspases (56). This activation leads to the cleavage of GSDMD at Asp276, generating GSDMD-NT, which oligomerizes and moves to the plasma membrane to form pores, ultimately inducing pyroptosis. This process can be inhibited by the chemotherapeutic drug oxidized 1-Palmitoyl-2-Arachidonoyl-sn-Glycero-3-Phosphocholine (oxPAPC) (57, 58). This distinctive mechanism may promote the selective recognition and interaction of Caspase with GSDMD, subsequently triggering its activation process (59). It is noteworthy that, unlike the direct effect of Caspase-1, the activation of Caspase-4/5/11 does not directly lead to the maturation of IL-1β and IL-18. However, the NLRP3 inflammasome can be activated by Caspase-11, promoting the maturation and release of IL-1β and IL-18 (60). This non-canonical pathway, by bypassing the inflammasome and not relying on its activation stimuli, provides an alternative regulatory mechanism for the inflammatory response. Additionally, Caspase-11 activation can trigger the canonical pyroptosis pathway by cleaving and modifying Pannexin-1, which crucially affects intracellular K^+^ efflux and ATP release. When ATP binds to the purinergic receptor P2X ligand-gated ion channel 7 (P2X7R), it opens the channel for Ca^2+^ and Na^+^ influx, causing rapid depolarization and subsequently triggering cellular pyroptosis (61, 62).

#### 2.2.3 Other pathways involved in pyroptosis

In their in-depth study of the mechanisms underlying pyroptosis, researchers identified granzymes as a class of serine proteases located in the cytosolic granules of cytotoxic T lymphocytes (CTLs) and natural killer cells (NK) cclls (63). Unlike the classical and non-classical pathways, Caspase-3, activated by granzyme B secreted by chimeric antigen receptor T cells and chemotherapy drugs, cleaves the GSDME protein, causing N-GSDME to migrate to the cell membrane and form pores, thereby initiating pyroptosis (64–66). Additionally, TAK1-activated Caspase-8 cleaves GSDMD, and granzyme A, released by lymphocytes, cleaves GSDMB within cancer cells; both processes culminate in pyroptosis (67, 68).

## 3 Pyroptosis and OA

OA is a chronic, aseptic inflammatory condition marked by degenerative alterations in cartilage and subsequent osteophyte formation. This disease clinically presents as joint pain, stiffness, swelling, and restricted mobility (69). Emerging research links the etiology and progression of OA to various components of the pyroptosis pathway, including inflammasomes, cytokines, and the Caspase family. This linkage suggests that inflammation and fibrosis, induced by pyroptosis in chondrocytes (70), are significant contributors to the disease mechanism. Further, current studies provide evidence of pyroptosis in both chronic aseptic inflammation and tissue fibrosis, highlighted by instances such as microglial pyroptosis inducing neuroinflammation and fibrosis (71) and renal macrophage pyroptosis leading to renal inflammation and fibrosis (72). The cytokines IL-1β and IL-18, produced during synoviocyte pyroptosis, exacerbate synovial inflammation and extracellular matrix (ECM) degradation. Conversely, inhibiting pyroptosis can mitigate synovitis and fibrosis in OA (73), revealing the pivotal role of chondrocyte and synoviocyte pyroptosis in the onse and progression of the disease.

### 3.1 Synovium and pyroptosis in OA

The synovium of OA exhibits a complex environment rich in various metabolites and soluble factors, which significantly contribute to the inflammatory responses and cartilage damage characteristic of OA (74). The intricate interaction between synovial tissue inflammation and pyroptosis mechanisms reveals that synovial tissue comprises fibroblast-like synoviocytes (FLS) and macrophages, exhibiting which substantial biological activity (75). These cells not only regulate the trafficking of small molecules but also play a pivotal role in mediating pyroptosis and inflammatory responses.

#### 3.1.1 FLS in the OA synovium

Within the joint space, synovial fluid is primarily produced by synovial tissue and secreted by FLS and macrophage. This fluid contains inflammatory mediators and cytokines, serving as a medium for intercellular material exchange (76). The synovial fluid facilitates interactions among released pro-inflammatory factors and proteases with various cellular components, triggering pyroptosis and contributing to progression changes in joint inflammation (73, 77). Notably, FLS are identified as the primary effector cells in synovial fibrosis. Further research indicates that multiple calcium salt crystals in the synovial fluid of OA patients may alter synovial permeability and decrease hyaluronic acid concentration. This alteration prompts FLS to secrete pro-inflammatory cytokines, thereby intensifying joint inflammation and damage (76, 78, 79).

During the development of synovitis, an extensive array of pro-inflammatory cytokines is released and transported into the synovial cavity. These cytokines serve not only as signaling molecules affecting the activities of various cells within the joint but also induce excessive and sustained inflammatory responses, which are primary contributors to OA and cartilage degeneration (70). HMGB1 is highly expressed in various inflammatory and autoimmune diseases, such as including sepsis (80), rheumatoid arthritis (RA) (81), and systemic lupus erythematosus (SLE) (82), acting as a DAMP to trigger inflammatory responses and exacerbatee disease progression. Research by Xiao et al. (83) demonstrates that in a rat model of knee osteoarthritis (KOA), significant increases in HMGB1 and fibrosis markers within the synovial tissue are due to pyroptosis in FLS, which in turn stimulates further production of these markers. Intriguingly, HMGB1 not only arises from pyroptosis but also feedbacks on FLS, enhancing the aggregation of pro-inflammatory factors and thereby exacerbating synovitis symptoms (84). The NLRP3 inflammasome plays a critical role in the synovial fluid of OA patients by facilitating the secretion of inflammatory cytokines that induce FLS pyroptosis (85). Under the combined influence of LPS and ATP, the expression of NLRP3-related proteins in FLS is significantly increased. In the KOA model, effectively inhibiting the NLRP3 signaling pathway reduces the expression of IL-1β, IL-18, and Caspase-1 (86). Activated IL-1β initiates pyroptosis in FLS and chondrocytes (CC), and stimulates the secretion of matrix-degrading enzymes crucial for the degradation of articular cartilage and cartilage damage (87). Zhang et al. (73) found that the number of NLRP3 inflammasomes increased in the synovial cells of OA rats. Knockout of Caspase-1 or the use of the Caspase-1 inhibitor Ac-YVAD-cmk could inhibit LPS-induced pyroptosis, as well as the expression of inflammatory markers and fibrosis. The NLRP3 inhibitor also reduces the expression of IL-1β, GSDMD, and Caspase-1 in synovial cells, indicating that both inhibitors significantly affect pyroptosis-related factors (88). Additionally, extracellular vesicles similar to exosomes play a crucial role in intercellular communication within joint tissues, impacting both the metabolism of the ECM and inflammatory processes (89). Wang et al. (90) discovered that miR-25-3p within fibroblast-like synoviocytes-extracellular vesicles (FLS-EVs), by binding to the cytoplasmic polyadenylation element-binding protein 1 (CPEB1), inhibits CPEB1 transcription. This inhibition leads to decreased expression of NLRP3, Caspase-1, GSDMD-NT, IL-1β, IL-18, and matrix metalloproteinases (MMPs), reducing CC pyroptosis and inflammation in mouse knee joints.

In summary, genetic engineering targeting or knocking out specific miRNAs in FLS-EVs, combined with the use of NLRP3 and Caspase-1 inhibitors to block interactions between inflammatory mediators such as HMGB1, IL-1β, IL-18, and matrix-degrading enzymes in synovial fluid, holds promise as a therapeutic approach for OA. This strategy is likely to become a focal point in future clinical research.

#### 3.1.2 Macrophage in the OA synovium

The macrophages possess phagocytic and immunomodulatory capabilities, which are responsible for producing chemotactic factors and cytokines that regulate inflammatory responses and promote tissue repair (91, 92). Synovial inflammation mediated by activated macrophages plays a significant role in the progression of OA. Upon the onset of OA, macrophages are exposed to stimulatory factors and exhibit distinct inflammatory properties. Pro-inflammatory macrophages exacerbate OA progression by infiltrating synovium and releasing excessive pro-inflammatory cytokines, which activate synovial fibrosis and further promote cartilage degradation (75). Thus, macrophages are pivotal in mediating intra-articular inflammation and maintaining tissue homeostasis, significantly influencing the pathogenesis of OA.

The functional plasticity of macrophages spans a polarization spectrum from pro-inflammatory M1 to anti-inflammatory M2 phenotypes, crucial for shaping the synovial inflammatory environment in OA (93–95). M1 macrophages promote inflammatory tissue damage by secretion of pro-inflammatory mediators such as IL-1β and TNF-α. Conversely, M2 macrophages mitigate immune hyperactivation and facilitate the resolution of inflammation through anti-inflammatory cytokines such as IL-10 and TGF-β, thus managing the balance between destructive and reparative phases in inflammatory diseases (96, 97). Research by Wang et al. (98) suggests that the elevated miR-146a levels in synovial exosomes of OA rates, compared to healthy counterparts, likely modulate the tumor necrosis factor receptor associated factor 6 (TRAF6)/NF-κB signaling pathway, thereby reducing CC degradation and affecting phenotypic transformation in synovial macrophage polarization. Additionally, macrophage supernatants stimulated by pentraxin 3 (PTX3) induces an OA phenotype in cartilage, demonstrating PTX3’s anti-chondrogenic effects mediated by macrophages. In the absence of miR-224-5p, increased PTX3 levels activate the p65/NF-κB pathway and promote M1 macrophage polarization. PTX3 also activates CD32 on macrophages, releasing pro-inflammatory mediators and further joint cartilage damage (99). The paracrine interactions between macrophages and chondrocytes create a feedback loop that exacerbates synovitis and cartilage damage. Therefore, modulating the polarization from M1 to M2 may represent a viable therapeutic strategy.

Emerging evidence emphasizes pyroptosis as critical mechanism in the functional transformation of macrophages, closely linked to intra-articular inflammation and the progression of OA. A study confirms that Caspase-1 siRNA transfected macrophages show decreased cell death in an LPS+ATP-induced pyroptosis model and co-culture with GSDMD siRNA transfected macrophages results in a significant reduction in FLS fibrosis marker levels (73). Additionally, research has pointed out that elevated chondrocyte inflammation in OA leads to cartilage degradation, with free cartilage fragments and released inflammatory mediators acting as DAMPs to trigger synovial macrophage pyroptosis, further exacerbating the inflammatory microenvironment and creating a vicious cycle (100). Moreover, a study by Xie et al. (101) demonstrated that Degrasyn, used in treating synovitis, reduces OA by inhibiting macrophage pyroptosis by suppressing the NLRP3/GSDMD signaling pathway.

In conclusion, M2 macrophages support chondrocytes in OA, revealing their therapeutic value (102). Future treatments may leverage exosomes or miRNA regulation to mitigate OA-related pyroptosis. These interventions aim to suppress the NLRP3 inflammasome and Caspase-1 activation in macrophages, inhibiting pyroptosis and M1 polarization while promoting M2 polarization. This strategy is anticipated to reduce IL-1β/IL-18 production, ultimately alleviating the progression OA.

### 3.2 Cartilage and pyroptosis in OA

Cartilage is a vital connective tissue in the human body, composed primarily of chondrocytes and the ECM. Encased within the ECM they themselves produce, CC are the sole cells capable of synthesizing and secreting both matrix and fibers, making them indispensable for sustaining a steady cycle of collagen metabolism (103). The ECM’s solid components predominantly include type II collagen (COL-II), proteoglycans, and minor amounts of other collagens and non-collagenous proteins (104). These elements are intricately woven to form a robust network, providing resistance to external mechanical pressures.

OA is primarily linked to chondrocyte necrosis, driven by various factors that disrupt the metabolic imbalance within chondrocytes and hinder the damaged cartilage’s self-repair capabilities. Several risk factors for OA, such as obesity (105), unhealthy lifestyles (106), lipopolysaccharides (107), hydroxyapatite crystals (108), and oxidized low-density lipoprotein (109), can initiate PAMPs or DAMPs. These, in turn, activate the NLRP3 inflammasome, leading to the release of proteolytic enzymes and inflammatory cytokines, including MMPs, a disintegrin and metalloproteinase with thrombospondin motifs (ADAMTS), IL-1β, IL-18, and tumor necrosis factor-α (TNF-α) (110). MMP13 and ADAMTS4/5 are recognized as key factors in ECM degradation, partially due to their upregulation by IL-1β (111).

The activation and assembly of the NLRP3 inflammasome also depend on a second signal, where ROS play a crucial role (112, 113). Studies have demonstrated that the activation of peroxisome proliferator-activated receptor gamma (PPAR-γ), primarily via the use of pioglitazone as an activator, markedly alleviates cellular oxidative damage induced by ROS, thus providing significant protection against chondrocyte damage and pyroptosis in OA (114, 115). Bai et al. have shown that activation of the adenosine A3 receptor (A3AR) with its agonist (CF101) effectively inhibits Caspase-1, offering protection to CC stimulated by hydrogen peroxide (H_2_O_2_). This protective effect is primarily achieved by blocking the ROS-induced activation of the NLRP3 inflammasome, highlighting the involvement of the NLRP3/Caspase-1 axis OA pathogenesis (116). Additionally, Zhang et al. (117) noted that the downregulation of miR-17 in OA chondrocytes of OA exacerbates pyroptosis progression. Conversely, supplementing exogenous miR-17 or inducing its endogenous expression through growth differentiation factor 5 can protect against OA by effectively inhibiting the activity of catabolic factors, including MMP3/13, ADAMTS5, and nitric oxide synthase 2 (NOS2). Similarly, the downregulation of miR-155 inhibits KOA in mice by targeting Smad2 and suppressing the NLRP3/Caspase-1 pathway, thus reducing pyroptosis in mouse KOA chongrocytes (118). Another study showed that exosomes from bone marrow mesenchymal stem cells (BMSC-Exos) specifically target histone deacetylase 3 (HDAC3) and the signal transducer and activator of transcription 1 (STAT1)/NF-κB pathway to inhibit pyroptosis in chondrocytes and cartilage tissue, while they also delivering miR-326 to chondrocytes, thus promoting the amelioration of OA (119).

Therefore, exploring the potential to inhibit chondrocyte pyroptosis presents a promising therapeutic strategy for halting ECM degradation and mitigating inflammatory responses in OA. This section summarizes the various inflammatory mediators released within the joint cavity during OA progression.

### 3.3 Subchondral bone and pyroptosis in OA

Subchondral bone, consisting of both bone matrix and bone cells, incorporates organic components such as collagen fibers and an amorphous matrix synthesized by osteoblasts. It also includes inorganic components, predominantly hydroxyapatite crystals, which provid hardness and toughness to the bone structure (120).

Studies on OA models have demonstrated that changes in subchondral bone are dynamic and often precede cartilage degeneration. These changes involve thickening the subchondral bone plate and trabecular bone remodeling, likely as an adaptation to altered joint loading (121). In mouse and rat OA models, early bone alterations such as trabecular reduction and osteosclerosis have been linked to the pyroptosis of articular chondrocytes (121, 122). Microstructural modifications in the subchondral bone influences its structure and integrity chondrocyte behavior, impacting biomechanical properties at various stages of OA. Microcomputed tomography (μCT) has revealed significant differences in the mechanical properties of subchondral bone between OA patients and healthy individuals, potentially exacerbating joint dysfunction (123). Initially, Chen et al. (124) observed reduced subchondral bone mass, increased trabecular separation, more osteoclasts, and decreased osteoprotegerin (OPG), indicating bone structure deterioration. Sun et al. (125) discovered that in a unilateral anterior crossbite mouse model, overexpression of miR-29b specifically in BMSCs, significantly alleviated cartilage degradation cartilage subchondral bone loss while reducing osteoclast overactivity. OPG plays a vital role in bone metabolism by inhibiting osteoclasts through binding to the NF-κB activating factor ligand (RANKL), and low levels OPG can disrupt bone balance, leading to increased resorption (126). Dihydroartemisinin (DHA), a derivative of artemisinin, effectively reduces osteoclast activity by targeting the NF-κB/MAPK/RANKL signaling pathway, thereby alleviating subchondral bone remodeling and associated cartilage degeneration in OA (127). Zheng et al. (128) conducted an experimental study on mice with destabilized medial meniscus (DMM) to demonstrate that paroxetine can inhibit pyroptosis and reduce osteoclast formation by suppressing the NF-κB/RANKL signaling pathway, suggesting its potential as a key therapeutic pathway in treating OA. The interplay between pyroptosis and osteoclast activity reveals the intricate mechanisms underlying OA pathology, where inflammatory cytokines released during pyroptosis may intensify bone degradation and contribute to the disease’s overall progression (100).

**Figure 2:**
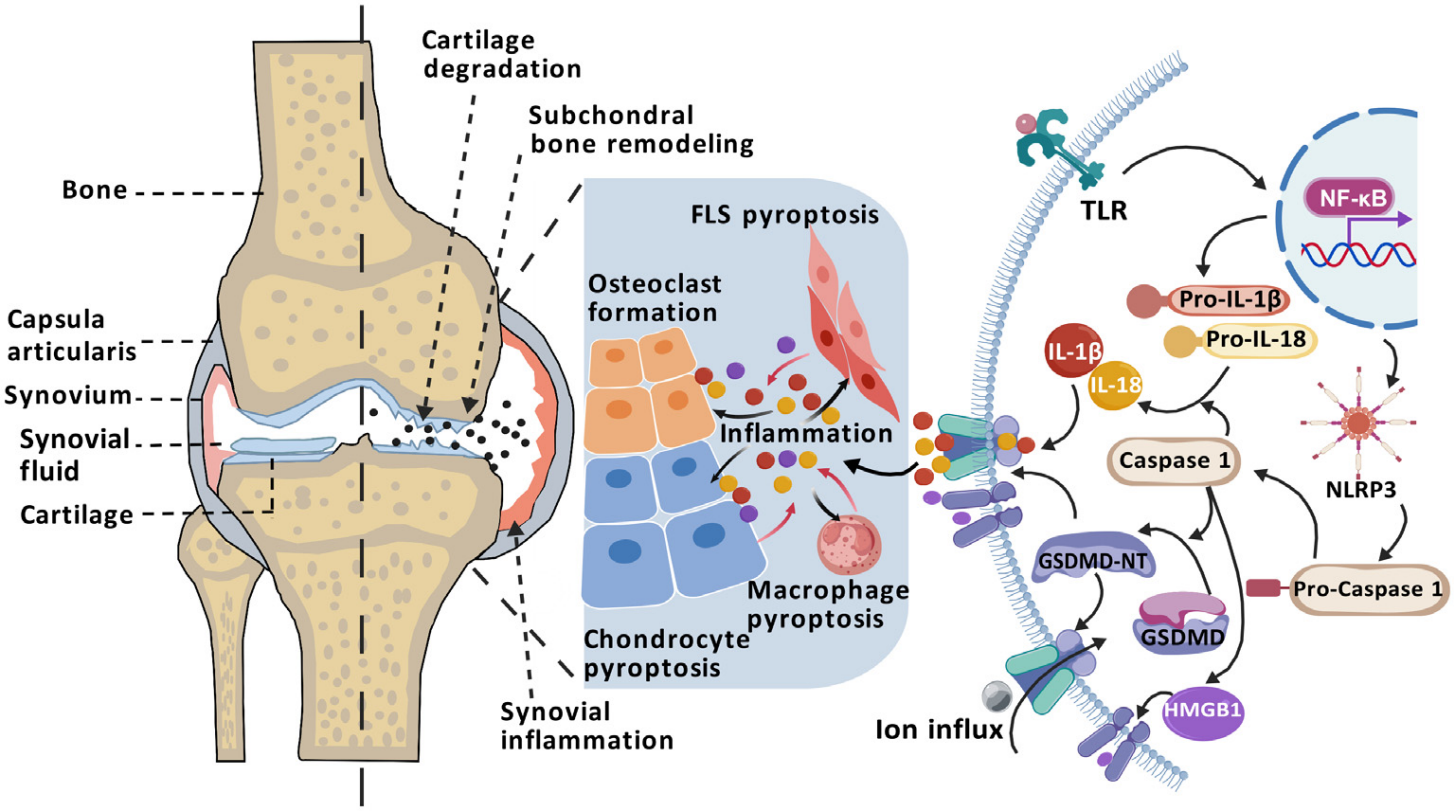
Mechanism of pyroptosis in OA pathogenesis. The progression of OA is closely associated with NLRP3 inflammasome-mediated pyroptosis. Activation of the TLR/NF-κB signaling pathway promotes the assembly of the NLRP3 inflammasome and activates Caspase-1, which cleaves GSDMD to generate the pore-forming GSDMD-NT, releasing inflammatory cytokines such as IL-1β, IL-18, and HMGB1. The accumulation of inflammatory mediators in the joint cavity—including IL-1β, IL-18, and HMGB1—promote FLS pyroptosis, macrophage pyroptosis, chondrocyte pyroptosis and osteoclast formation. Consequently, in a persistent chronic inflammatory environment, it can cause synovial inflammation, osteophyte formation, cartilage degradation, leading to OA. (Created with BioGDP.com).

Indeed, a rigorous exploration of the interactions between macrophages, FLS, CC, and pyroptosis is vital for advancing our comprehension of the pathophysiological mechanisms underlying OA. Such studies are poised to inform the creation of innovative therapeutic strategies and methodologies. The interactions among macrophages, FLS, and CC, closely tied to pyroptosis, are likely crucial drivers of OA progression at various disease stages—whether early, intermediate, or late—and may impact the disease trajectory collectively or independently. The role of the NLRP3 inflammasome and its downstream factors in disrupting the microenvironments of different tissue cells during the OA progression has garnered increasing attention (**Figure 2**). Unfortunately, comprehensive studies investigating the specific roles of pyroptosis at different stages of OA are lacking. Current research primarily utilizes imaging technologies and histological techniques to detail histopathological changes and inflammasome-related molecular features in end-stage OA. These studies do not capture the dynamic changes throughout the disease course and fail to offer definitive conclusions regarding the controll of cell pyroptosis at various stages. Consequently, it is critically important to thoroughly understand the specific roles and intricate mechanisms of pyroptosis in the interactions among different cell types at various OA stages. Such knowledge could accelerate the development of more targeted interventions, aiming to halt or even reverse the pathological processes of OA from onset.

Furthermore, miRNAs play a crucial role not only in regulating the pyroptosis of macrophages, FLS, and CC, but also in influencing the pyroptosis of subchondral bone cells during the progression of OA (129, 130). Recent evidence suggests that inappropriate macrophage polarization responses are pivotal in the damage to bone and joint tissues. Given this, miRNAs are increasingly recognized as important upstream regulators of pyroptosis, and numerous new studies have confirmed that miRNAs can modulate the polarization between macrophages phenotypes in such contexts. Additionally, the balance between pyroptosis of non-immune cells and the inflammatory microenvironment in joints results from regulation by miRNAs. This understanding has led to the strategy of using miRNAs within vesicles secreted by intra-articular tissue cells to precisely regulate the activation pathways of NLRP3 inflammasomes, regarded as a promising therapeutic approach for treating osteoarthritic diseases. These miRNA regulatory pathways in the pyroptosis mechanism are summarized in **Table 1**.

**Table 1:** Summary the targeting miRNAs to regulate mediated pyroptosis in OA.

Target gene	Possible signaling Pathway	Biological function	Reference
miR-665	CircRNF121/MYD88/NF-κB	Inhibit cartilage matrix degradation and oxidative stress	(45)
miR-25-3p	miR-25-3p/CPEB1	Alleviate the pyroptosis of cartilage	(90)
miR-146a	Toll4/TRAF6/NF-κB	Regulates cartilage degradation and macrophage polarization	(98)
miR-224-5p	p65/NF-κB	Regulates macrophage reprogramming and exacerbates synovitis	(99)
miR-326	HDAC3 and STAT1/NF-κB/NLRP3	Inhibit CC pyroptosis	(119)
miR-155	Smad2/NLRP3/Caspase-1	Inhibit inflammation and pyroptosis	(118)
miR-17	miR-17/HIF-1α	maintaining cartilage homeostasis and protection against OA	(117)
miR-29b	miR-29b/Wnt5a	Promotes subchondral bone loss	(125)

## 4 The treatment of OA pyroptosis

### 4.1 Targeting NLRP3 for the treatment of pyroptosis

The inflammasome, a complex structure integral to the immune response, consists of core proteins such as NLRP1, NLRP3, NLRC4, AIM2, pyrin, and IFI16 (131), with NLRP3 being particularly significant due to its involvement in numerous pathophysiological responses. The NLRP3 inflammasome can be activated through various stimuli, including LPS, ROS, ATP, Ca^2+^ inflow, K^+^ outflow, inflammatory factors, lysosomal instability, mitochondrial dysfunction, and diverse pathogens (46, 132, 133). Research has highlighted an increased activation of NLRP3-related proteins in OA patients, and LPS-induced pyroptosis is alleviated by NLRP3 siRNA (134).

In cases of temporomandibular joint osteoarthritis (TMJOA), the use of Caspase-1 inhibitor Ac-YVAD-cmk and the NLRP3 inhibitor MCC950 has shown effectiveness in inhibiting LPS-induced pyroptosis and reducing inflammation and fibrosis (88). Moreover, the ubiquitination of thioredoxin-interacting protein (TXNIP) by the USP family leads to ROS accumulation, activating the NLRP3 inflammasome, increasing IL-1β and IL-18 production, and promoting GSDMD-N-dependent pyroptosis and ECM remodeling (135). The role of guanylate binding protein 5 (GBP5), a member of the Interferon-gamma-induced guanosine triphosphatase family, is also noteworthy. In TNF-α-induced models, GBP5 expression is upregulated, inhibiting the synthesis of ECM and COL-II and promoting the expression of NLRP3, Caspase-1, GSDMD, and MMP13. Additionally, IRF1 is found to bind to the promoter region of GBP5, thereby enhancing its expression (136). The involvement of O-GlcNAc transferase (OGT)-induced O-linked N-acetylglucosamine (O-GlcNAcylation) in many human diseases has been documented, though its specific role in OA remains unclear. Research by He et al. (137) indicates that the silencing or knockout of OGT increases the phosphorylation of (Never in Mitosis Gene A)-related kinase 7 (NEK7), particularly at the S260 residue. This phosphorylation impedes the interaction between NEK7 and NLRP3, effectively suppressing LPS-induced chondrocyte pyroptosis. Furthermore, pharmacological agents such as the NLRP3 inhibitor CY-09 (138), icariin (139) and Sipeimine (Sip) (140) have demonstrated efficacy in decreasing NLRP3 inflammasome expression, inhibiting chondrocyte pyroptosis, reducing catabolic and inflammatory responses, and ameliorating cartilage damage.

Therefore, inhibition of NLRP3 activity or blockade of its upstream signaling pathway may reduce the joint inflammatory response, protect chondrocytes, and delay the progression of OA. Further investigation into the mechanism of NLRP3 in OA is expected to provide novel targets and approaches for OA treatment (**Table 2**).

**Table 2:** Summarize the regulatory factors involved in NLPR3-mediated pyroptosis in OA.

Regulatory element	Signaling Pathway	Biological function	Animal models	Reference
Degrasyn	NLRP3/GSDMD	Inhibits synovial macrophagic pyroptosis	Mice	(101)
CF101	ROS/NLRP3/GSDMD	Inhibits OA progression and relieves pain perception	Rats	(116)
Ac-YVAD-cmk or MCC950	NLRP3/Caspase-1	Inhibit pyrodeath, inflammation and fibrosis	Rats	(88)
USP25	TXNIP/ROS/NLRP3	Promotes cartilage cell damage	Rats	(135)
OGT	NEK7/NLRP3	Inhibits cartilage degradation	Mice	(136)
IRF1/GBP5	IRF1/GBP5/NLRP3	Accelerate cartilage degradation	Mice	(137)
CY-09	NLRP3	Inhibits cartilage degradation	Rats	(138)
Icariin	NLRP3/Caspase-1	Inhibits inflammation and pyroptosis	Rats	(139)
Sip	PI3K/AKT/NF-κB/NLRP3	Alleviate the subchondral remodeling, synovitis as well as ECM degradation	Mice	(140)

### 4.2 Targeting NF-κB for the treatment of pyroptosis

Nuclear factor kappa-B (NF-κB) is an essential family of nuclear transcription factors that includes RelA, RelB, c-Rel, p52/p100, and p50/p105. These factors are vital for processes such as inflammation, immune response, and cell proliferation (141). They regulate gene expression by forming dimers with dynamic compositions. Notably, the RelA-p52 heterodimer plays a crucial role in late NF-κB activation, bridging classical and non-classical pathways (142).

The activation of the NF-κB signaling pathway generally depends on a diverse array of intracellular stimuli in articular tissues, such as cytokines, oxidative stress, I-κB phosphorylation, and motor stimulation, which typically leads to pyroptosis (143). These stimuli activate the inhibitor of the kappa B kinase (IKK) complex, resulting in IκB degradation and enabling NF-κB to enter the nucleus, bind DNA, and regulate gene transcription. NF-κB also collaborates with other transcription factors to refine its regulatory role, and it activates NLRP3, which promotes inflammasome assembly, inflammation, and pyroptosis (144). Numerous studies (145–148) have demonstrated that specific small molecule drugs, including Ursolic acid, GYY4137, Loganin, and α-Solanine, can reduce Caspase-1 and MMP protein levels and enhance COL-II expression by decreasing IκBα phosphorylation and blocking p65 nuclear translocation. Furthermore, inhibiting Caspase-1 and GSDMD activity, along with preventing ECM degradation and pyroptosis, effectively alleviates LPS-induced OA symptoms. Additionally, compounds such as Resolvin D1, derived from omega-3 fatty acids, and spermidine (SPD), exhibit anti-inflammatory effects in chondrocytes by inhibiting the NLRP3/Caspase-1/GSDMD pathway (149, 150). This restoration of COL-II expression, reduction in MMP13 and ADAMTS5 levels, improvement in chondrocyte viability, and decrease in pyroptosis contribute significantly to therapeutic outcomes.

In bone metabolism research, NF-κB plays a dual role by influencing both osteoclast formation and osteoblast function. The MEK inhibitor PD0325901 has demonstrated significant effectiveness in inhibiting the increase in NF-κB and NLRP3 levels that result from subchondral destruction (151). Moreover, small mechanical stress can inhibit pyroptosis by activating transforming growth factor-β (TGF-β) and Smad2/3 while concurrently suppressing the NF-κB/NLRP3 pathway. This dual action reduce inflammation and osteoclas activity, thereby aiding in the reconstruction of subchondral bone. Similarly, the knockdown of miR-155 achieves comparable effects through the Smad2/NF-κB/NLRP3 pathway (118, 143, 152).

In summary, the NF-κB/NLRP3 signaling pathway is central to numerous biological processes and pathologies. An enhanced understanding of its mechanisms could provide novel insights and strategies for treating OA, as outlined in **Table 3**.

**Table 3:** Summarize the regulatory factors involved in NF-κB-mediated pyroptosis in OA.

Regulatory element	Signaling Pathway	Biological function	Animal models	Reference
Mechanical stress	TGF-β/Smad2/3/NF-κB	Inhibit chondrocyte pyroptosis	Rats	(143)
Ursolic acid	NF-κB/NLRP3	Inhibits cartilage degradation	Rats	(145)
GYY4137	NF-κB/NLRP3	Inhibits synovial macrophagic pyroptosis	Mice	(146)
Loganin	NF-κB/Caspase-1	Inhibits cartilage degradation	Mice	(147)
α-Solanine	NF-κB/NLRP3	Reduces osteophyte formation and subchondral sclerosis	Mice	(148)
Resolvin D1	NF-κB/NLRP3/Caspase-1	Promotes chondrocyte proliferation and repair	Rats	(149)
SPD	NF-κB/NLRP3/Caspase-1	Promote the cartilage integrity and suppress ECM	Rats	(150)
PD0325901	NF-κB/NLRP3	Inhibits subchondral bone destruction	Mice	(151)
Less mechanical loading	NF-κB/NLRP3	Inhibits cartilage degradation, subchondral bone remodeling	Rats	(152)

### 4.3 Targeting P2X7R for the treatment of pyroptosis

P2X7R is an ATP-sensitive ion channel characterized by an intracellular amino terminus, a carboxyl terminus, and two hydrophobic transmembrane segments separated by glycosylated extracellular ATP-binding domains (153). In the pathological context of OA, P2X7R also mediates the influx of Na^+^ and Ca^2+^ and the efflux of K^+^, contributing to various inflammatory responses and playing a significant role in different mechanisms of cell death (61, 154).

Due to cell damage and inflammatory responses in OA, the release of ATP is crucial in activating P2X7R. This activation enhances NLRP3 inflammasome activity, fostering inflammatory responses and potentially inducing pyroptosis (155, 156). P2X7R acts as a key regulator of inflammation by modulating the ionic environment within and outside the cell, promoting the assembly and activation of the NLRP3 inflammasome. This activation results in the release of pro-inflammatory cytokines such as IL-1β and IL-18, thereby intensifying joint inflammation and damage (156–158). Research indicates that the activation of the NLRP3 inflammasome is closely linked to the modulation of the adenylate-activated protein kinase (AMPK)/mechanistic target of the rapamycin (mTOR) signaling pathway. Decreased AMPK activity as a cellular energy sensor may lead to NLRP3 overactivation. Conversely, linear ubiquitinated LKB1 can activate the AMPK pathway, reversing the NLRP3 inflammasome response and potentially halting the progression of pyroptosis (159, 160). Additionally, Liang et al. found that piperine, a pharmacologically active phytochemical found in black pepper with anti-inflammatory properties, can significantly inhibit ATP-induced AMPK activation, thereby markedly suppressing pyroptosis and reducing IL-1β levels (161). Activation of AMPK inhibits the mTOR signaling pathway, which plays a critical role in regulating cell growth, promoting autophagy, eliminating excess or damaged cellular components, and maintaining cellular health (162). Experimental evidence indicates that high expression of mTOR suppresses autophagy in articular CC, accelerating cartilage degeneration. Conversely, inhibition of mTOR activity can trigger autophagy, reducing pyroptosis and cartilage loss (163). Research has demonstrated that mTOR inhibitors, such as rapamycin and the traditional Chinese medicine Gu yan xiao tincture, as well as the P2X7R inhibitor A740003, effectively inhibit mTOR, induce autophagy, and reduce pyroptosis and cartilage loss. On the other hand, injection of mTOR agonists, such as MHY1485, or P2X7R agonists, such as Bz-ATP, promotes pyroptosis and ECM degradation (163–166). Additionally, during moderate-intensity exercise, P2X7R activation facilitates ion flux, which activates the AMPK/mTOR signaling pathway. This promotes autolysosomal targeting and degradation of the inflammasome component NLRP3, thereby inhibiting autophagy and alleviating pyroptosis (167).

Consequently, the P2X7R functions as a pivotal ion channel, modifying the ionic milieu upon ATP stimulation and facilitating the production of NLRP3. The P2X7R can initiate both autophagy and pyroptosis. Engaging in early and appropriate physical activity can stimulate autophagy through the P2X7R, potentially slowing the progression of OA, though as the condition evolve, it may towards pyroptosis (167). The extent of P2X7R activation closely correlated with cellular destiny, and suggesting that judicious activation may hold the key to effective OA treatment, as outlined in **Table 4**.

**Table 4:** Summarize the regulatory factors involved in P2X7R-mediated pyroptosis in OA.

Regulatory element	Possible signaling Pathway	Biological function	Animal models	Reference
A740003	P2X7R	Inhibiting ATP-induced pyroptosis	Mice	(62)
ATP	P2X7R/Caspase-3/7	Inhibiting cartilage damage	Rabbits	(155)
Linear Ubiquitination of LKB1	AMPK/NLRP3	Ameliorated inflammasome response and chondrocyte injury	Rats	(160)
Piperine	ATP/AMPK/mTOR	Inhibition of macrophage pyroptosis	Mice	(161)
Rapamycin	mTOR	Inhibit autophagy of articular chondrocytes	Mice	(163)
Gu yan xiao tincture	mTOR	Alleviate tissue damage in rabbit models of OA	Rabbits	(164)
MHY1485	mTOR	Promote ECM degradation and cartilage injury	Mice	(165)
Moderate exercise	P2X7/AMPK/mTOR	Promote autophagy in OA to alleviate pyroptosis	Rats	(167)

### 4.4 Targeting HIF-1α and Nrf2/HO-1 for the treatment of pyroptosis

HO-1 is critical for heme catabolism, providing both antioxidant and anti-inflammatory protection. Nrf2 is a key transcription factor that upregulates the expression of HO-1, enhancing Nrf2 activity and creating a positive feedback loop. During hypoxia, the activation of HIF-1α promotes the expression of HO-1, thereby strengthening the cellular antioxidant capacity and forming a robust feedback mechanism under hypoxia and oxidative stress (168, 169). The role of HIF-1α in the tumor and inflammatory microenvironments is intricately linked to the Nrf2/HO-1 pathway, exerting a complex influence on cell proliferation and survival (170). Research has shown that compounds such as PD184352 and Myrislignan activate the Nrf2/HO-1 signaling pathway, promoting the expression of antioxidant proteins, reducing the accumulation of ROS, and ultimately inhibiting the production of IL-1β-induced nitric oxide (NO), inducible nitric oxide synthase (iNOS), and Prostaglandin E2 (PGE2), while also attenuating pyroptosis (171, 172).

The bone-cartilage interface, particularly the calcified cartilage, connects firmly to the subchondral bone, preventing blood vessels from invading the hyaline cartilage (173, 174). Activation of HIF-1α upregulates the expression of VEGF, promoting angiogenesis and infiltration into calcified cartilage areas, which alters the microenvironment of articular cartilage and leads to tissue damage (175). Feng et al. (176) conducted a study in which human synovial fibroblasts were stimulated with HMGB1, resulting in elevated expression levels of HIF-1α and VEGF. This finding further substantiates the pivotal role of HIF-1α in the pathogenesis of OA. Similarly, Zhang et al. (177) observed a significant increase in HIF-1α activity within the synovial tissue of OA rat models. Furthermore, silencing HIF-1α reduced the expression of fibrosis-related markers, including TGF-β, type 1A1 collagen (COL-1A1), and tissue inhibitor of metalloproteinases-1 (TIMP1). Further studies have demonstrated that the natural small molecule AGN, derived from verbena family extracts, inhibits the HIF-1α/NLRP3 signaling pathway. This inhibition reduces LPS-induced levels of NLRP3, IL-1β, and IL-18, contributing to reduced hypoxia, inflammation, and fibrosis in synovial tissue (178). Zhang et al. (130) discovered that lymphocyte cytosolic protein 1 (Lcp1) knockout in ACLT mice inhibited osteoclast activation in subchondral bone. This inhibition subsequently decreased HIF-1α levels, reduced subchondral bone remodeling, and slowed cartilage degeneration, alongside an increase in H-type vessels and oxygen concentration. Additionally, they also found that Oroxylin A, an inhibitor of the Lcp1-encoded protein l-plastin (LPL), could alleviate the progression of OA.

In summary, using HIF-1α-specific inhibitors or gene knockout can alleviate OA induced by various fibrotic markers, whereas the Nrf2/HO-1 pathway can also inhibit the inflammatory progression of OA and reduce fibrotic indicators. However, HIF-1α can also exacerbate OA by promoting the release of fibrotic indicators such as TGF-β, COL-1A1, and TIMP1, which are activated by VEGF. Therefore, the dual roles of HIF-1α need to be utilized judiciously, and more extensive and precise research is required to elucidate the relationship between these pathways, as outlined in **Table 5**.

**Table 5:** Summarize the regulatory factors involved in Nrf2/HO-1 and HIF-1α-mediated pyroptosis in OA.

Regulatory element	Signaling Pathway	Biological function	Animal models	Reference
Oroxylin A	Lcp1/HIF-1α	Decreased subchondral bone remodeling and slowed cartilage degeneration	Mice	(130)
PD184352	Nrf2/HO-1	Anti-inflammatory and antioxidant effects	Mice	(171)
Myrislignan	Nrf2/HO-1	Inhibits inflammation and oxidative stress	Rats	(172)
HMGB1	HO-1/VEGF	Inhibits synovial angiogenesis	Human FLS	(176)
Silence HIF-1α	HIF-1α/NLRP3	Inhibit pyroptosis of FLS	Rats	(177)
Agnuside(AGN)	HIF-1α/NLRP3	Relieves synovitis and fibrosis in KOA	Rats	(178)

### 4.5 Cellular therapy

Cellular therapy, a therapeutic strategy utilizing specific cells and their functions for tissue repair, has shown promising prospects in treating OA recently. The core mechanisms of this approach include the inhibition of inflammation, regulation of cell death, and promotion of tissue regeneration. Recent research has demonstrated that human adipose-derived mesenchymal stem cells (hAD-MSCs) can significantly delay in rats and improve joint pathology by suppressing the expression of NLRP3, Caspase-1, GSDMD, and TNF-α receptor 1 (TNFR1). Further *in vitro* experiments have validated that the abundant soluble TNF-α receptor 1 (sTNFR1) secreted by hAD-MSCs competes with TNFR1 on the surface of chondrocytes for TNF-α binding. This competition inhibits chondrocyte pyroptosis (179), highlighting the therapeutic potential of hAD-MSCs, particularly in targeting chondrocyte pyroptosis. By modulating the NLRP3 pyroptosis pathway, cellular therapy effectively inhibits the release of inflammatory cytokines and cartilage destruction, offering a novel perspective for OA treatment and infusing new vitality into its clinical application.

Future research may explore pretreatment strategies to enhance the therapeutic potential of seed cells, aiming to achieve efficient intervention in pyroptosis and rapidly restore the balance of pyroptosis in OA.

### 4.6 Exercise and diet therapy

OA is a degenerative joint disorder characterized by the progressive loss of cartilage, resulting in pain and functional impairment. Recent research has highlighted the potential of dietary therapies, such as the ketogenic diet (180), and physical therapies, including exercise therapy (181) and reducing mechanical load on the knee joint (152), in influencing the pathogenic mechanisms of CC pyroptosis in OA. This connection raises important questions about how interventions such as exercise and dietary adjustments can affect cellular processes involved in the management of OA.

Exercise has been shown to play a dual role in OA management. Moderate physical activity generally benefits joint health by activating various cell signaling pathways, promoting cartilage health and reducing inflammation. Conversely, excessive or inappropriate exercise may increase cell pyroptosis, exacerbating cartilage degeneration (143, 181, 182). A systematic review and meta-analysis have revealed the benefits of therapeutic exercise, demonstrating significant improvements in pain and physical function compared to non-exercise controls. The findings from these studies suggest that the effects of exercise therapy are particularly beneficial for individuals with shorter symptom durations, indicating a critical window for intervention (183). Controlled exercise interventions that normalize joint movement have been shown to mitigate cartilage degeneration, suggesting that tailored exercise regimens could enhance the effectiveness of treatment for OA (184). Similarly, dietary interventions play a crucial role in managing OA symptoms. Research indicates that the intake of specific polyunsaturated fatty acids (PUFAs) can modulate inflammation and improve cartilage health. For instance, a study found that a diet low in n-6/n-3 PUFA significantly improved cartilage structure and inhibited articular cartilage polysaccharide loss in osteoporotic mice, highlighting the potential of dietary strategies in OA management (185). Furthermore, dietary therapy affects CC pyroptosis within the joint, which is a key mediator of pain and functional limitation in OA. Jin et al. (186) discovered that dietary fatty acids (FAs) are intimately linked to CC pyroptosis in obese mouse models of post-traumatic OA and LPS-stimulated CC, potentially by modulating the TLR4 signaling pathway to affect the NLRP3/Caspase-1/GSDMD pathway. Notably, a diet abundant in PUFAs can alleviate OA and reduce pyroptosis. Therefore, the cell pyroptosis pathway may be a crucial mechanism underlying the therapeutic effects of exercise and dietary interventions for OA. This provides a theoretical foundation for such treatment interventions.

**Figure 3:**
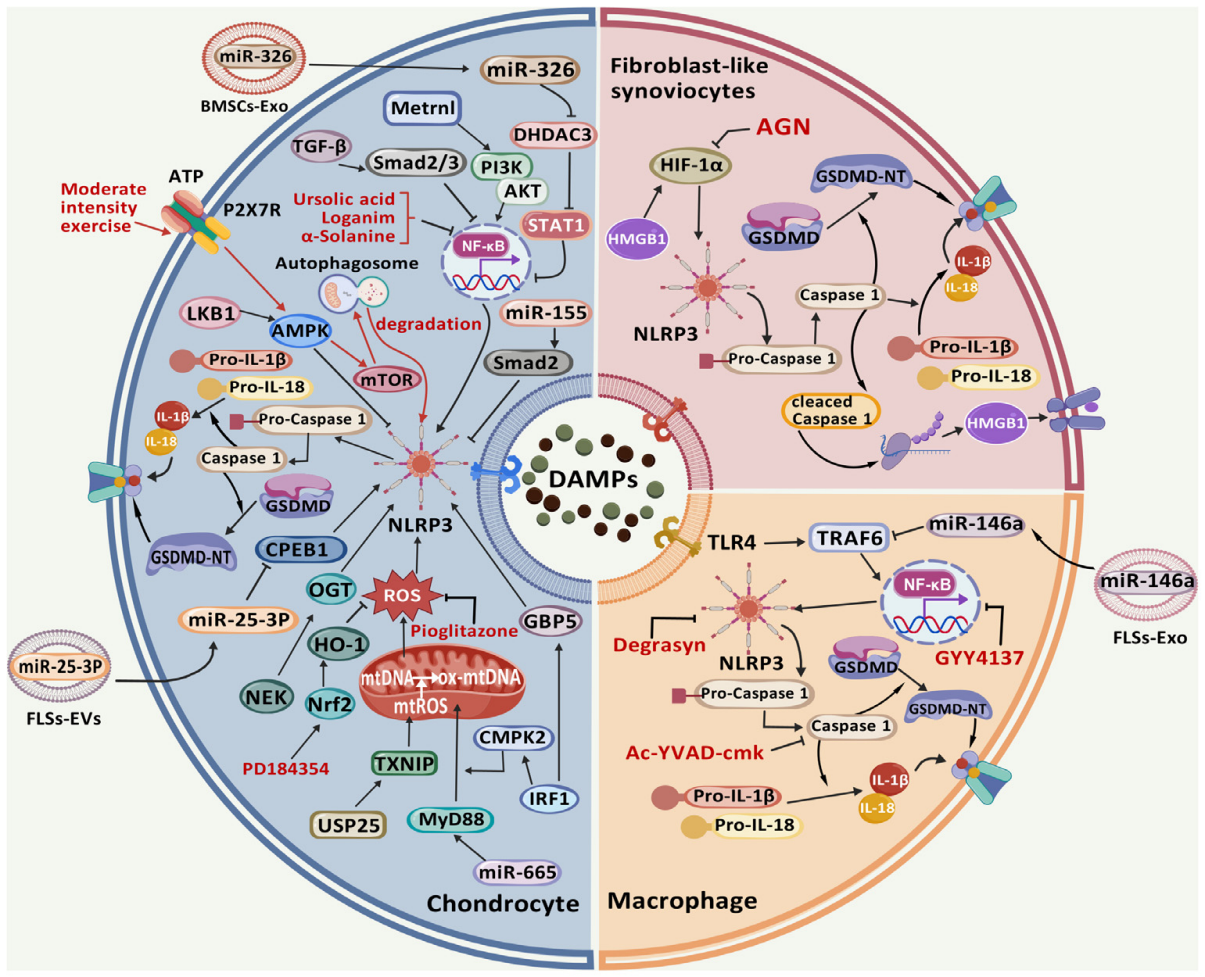
The signaling pathways involved in the pathophysiological development of OA, along with their corresponding targeted therapeutic drugs. During OA progression, pyroptosis in articular tissue cells is regulated through multiple molecular targets. Exposure to PAMPs or DAMPs initiates TLR-mediated activation of the NLRP3 inflammasome and Caspase-1. Subsequently, Caspase-1 and Caspase-4/5/11 cleave GSDMD to generate GSDMD-NT that oligomerize on plasma membranes, forming transmembrane pores. This process facilitates the proteolytic maturation and release of pro-inflammatory cytokines IL-1β and IL-18, ultimately triggering pyroptosis. The NLRP3 inflammasome activation is potentiated through ROS accumulation mediated by USP25/TXNIP and miR-665/MyD88 axis, which can be pharmacologically suppressed by pioglitazone and PD184352 via Nrf2/HO-1 signaling. Multiple regulatory pathways including IRF1/GBP5, NEK/OGT, miR-25-3P/CPEB1, miR-155/Smad2, and LKB1/AMPK converge on NLRP3 to modulate CC pyroptosis. Additional modulators such as miR-326/HDAC3/STAT1, Metrnl/PI3K/AKT, TGF-β/Smad2/3 signaling, along with pharmacological agents ursolic acid, loganin, and α-solanine, exert regulatory effects through NF-κB/NLRP3 pathway manipulation. Mechanical and biochemical stimuli demonstrate protective potential: ATP and moderate intensity exercise activate P2X7R ion channels, witch subsequently activate AMPK/mTOR-mediated autophagy for NLRP3 inflammasome component degradation. HMGB1 and AGN regulate FLS pyroptosis through HIF-1α/NLRP3 signaling. Macrophage pyroptosis is modulated by GYY4137 via NF-κB/NLRP3 axis, while pharmacological inhibitors Degrasyn and Ac-YVAD-cmk specifically target NLRP3 and Caspase-1 respectively to suppress this process. (Created with BioGDP.com).

In conclusion, integrating of therapeutic medications targeting the pyroptosis signaling pathway in OA with exercise and dietary interventions constitutes a robust foundation for holistic OA management. It is anticipated that these strategies will undergo continuous refinement and expansion, ultimately benefiting a broader spectrum of OA patients (**Figure 3**).

## 5 Summary and outlook

In recent years, an increasing body of research has established a significant link between pyroptosis, a pro-inflammatory programmed cell death pathway, and the pathogenesis of OA, particularly concerning articular cartilage and synovial tissue lesions. Consequently, this has led to a detailed exploration of various signaling pathways and targeted therapeutic drugs that may influence the development of OA. Extensive documentation has revealed changes in inflammatory mediators during the pathological progression of OA, including members of the Caspase family, IL-1β, IL-18, TNF-α, HMGB1, and proteins such as MMP and ADAMTS. A notable finding is a positive correlation between the levels of NLRP3-mediated pyroptosis in OA patients’ cartilage and the severity of the disease (187). *In vitro* and animal model studies have shown a significant correlation between the suppression of molecules associated with pyroptosis, and the subsequent alleviation of OA symptoms. However, despite the mounting evidence of pyroptosis’s critic role in OA, there is still a shortage of OA-specific regulatory mechanisms identified, and more specific drugs targeting pyroptosis are needed. Moreover, evaluating the mechanisms of the potential adverse effects of these treatments is essential to avoid osteolytic inflammatory responses and enhance the therapeutic effectiveness. Specifically, drug application strategies must balance defensive pyroptosis to prevent antagonistic interference between different factors.

Research has highlighted the central role of the NLRP3 inflammasome in the pathogenesis of osteoarthritic diseases. Inhibiting its activation can significantly reduce the inflammatory response in OA and improve the local pathological condition of the joint. Given the complex interactions among multiple cell types within the joint, treating of OA should not be limited to targeting a single area or cell type. Indeed, adopting a comprehensive treatment approach and emphasizing the synergistic effects among cells is crucial. Investigating the interactions between cells and pyroptosis within joint tissues will enhance our understanding of the pathophysiological processes of OA. Whether through regulating CC, macrophages, FLS, cellular therapy, or adopting exercise and dietary therapies, most current methods likely alleviate or inhibit pyroptosis in OA by activating or inhibiting various signaling pathways such as NF-κB, MAPK, P2X7R, Nrf2/HO-1, and HIF-1α, which are activated by the NLRP3 inflammasome. Notably, while numerous studies indicate that the occurrence of pyroptosis accelerates the progression of OA, a few studies have expressed skepticism about the positive effects of inhibiting pyroptosis in the synovium, highlighting the need for further research to clarify these potential discrepancies and develop more targeted and effective treatments (188). More extensive research is needed to elucidate the upstream or downstream pathways and key targets of the NLRP3 inflammasome, further enhancing our understanding of its precise regulation and deeper pathological significance, which will contribute to providing more options for the prevention and treatment of human OA. On the other hand, as elaborated in previous sections, the spatiotemporal regulation of pyroptosis across various OA stages may present a promising therapeutic potential. By integrating single-cell spatial transcriptomics to map pyroptosis-associated gene networks within joint microenvironments, this approach could identify stage-specific biomarkers, enabling precision therapies that are dynamically aligned with OA progression. Such strategies may address current limitations in temporally adaptive interventions, shifting the paradigm from broad inflammasome inhibition to context-dependent regulation of pyroptosis.

In summary, current research on OA primarily focuses on cellular and animal models, with relatively few studies involving clinical patients. The inherent limitations of these models in replicating the complex human microenvironment reduce the persuasiveness of these studies. Before progressing to the clinical trial phase, a thorough assessment of the treatment’s safety and potential risks must be conducted to ensure that patient health and safety are not compromised. Alternatively, there is growing interest in the emergence of targeted molecular biomaterials in the therapeutic landscape of OA, which have shown exceptionally promising prospects for clinical translation and application. These materials, including extracellular vesicles, hydrogels, and scaffolds, highlight the significant potential in advancing OA treatment strategies (189). Looking forward, it is crucial to further explore the molecular mechanisms of pyroptosis and its role in OA, seeking effective therapeutic strategies and potential targets. The combination of pyroptosis-targeting drugs with targeted biomaterials aims to achieve precision treatment and ultimately cure patients, marking an exciting direction in the field of OA treatment.
